# CHANGE IN UNMET NEEDS STATUS FOR DAILY MOBILITY AND SELF-CARE ACTIVITIES

**DOI:** 10.1093/geroni/igae098.1827

**Published:** 2024-12-31

**Authors:** Laura Sands, Xiaofan Zhu, Maham Khan, Pang Du

**Affiliations:** Virginia Tech, Blacksburg, Virginia, United States; Virginia Tech, Blacksburg, Virginia, United States; Virginia Tech, Blacksburg, Virginia, United States; Virginia Tech, Blacksburg, Virginia, United States

## Abstract

Unmet need occurs when older adults with difficulties completing daily activities cannot complete an activity because no one is available to help. Prior research has not considered whether unmet need status changes over time, nor assessed characteristics of older adults whose unmet needs worsen or resolve. Changes in unmet needs status across three pairs of consecutive annual interviews were assessed using 2011-2014 data from the National Health and Aging Trends Study. Findings revealed that that 57% of older adults who needed help with mobility activities and 62% of older adults who needed help with self-care activities did not experience unmet needs across the three consecutive pairs of occasions. Only 3% who needed help with mobility activities and 1% who needed help with self-care activities consistently reported unmet needs. Worsening of mobility unmet needs occurred for 25% and 16% resolved their mobility unmet needs. Worsening of self-care unmet needs occurred for 25% and 11% resolved their self-care unmet needs. Those who experienced worsening of mobility and/or self-care unmet needs were more likely to be female and have multiple chronic conditions. Those who experienced resolution of mobility unmet needs were more likely to be female and have multiple mobility difficulties and chronic conditions. Those who experienced resolution of self-care unmet needs were more likely to be white race, have zero or one self-care difficulties, and have multiple chronic conditions. The findings inform the need for frequent assessment of unmet needs to identify opportunities to reduce worsening and increase resolution of unmet needs.

